# Role of the ATPase/helicase maleless (MLE) in the assembly, targeting, spreading and function of the male-specific lethal (MSL) complex of *Drosophila*

**DOI:** 10.1186/1756-8935-4-6

**Published:** 2011-04-12

**Authors:** Rosa Morra, Ruth Yokoyama, Huiping Ling, John C Lucchesi

**Affiliations:** 1 Department of Biology, Emory University, 1510 Clifton Road, Atlanta, GA 30322, USA; 2 Paterson Institute for Cancer Research, The University of Manchester, Manchester, M20 4BX, UK

## Abstract

**Background:**

The male-specific lethal (MSL) complex of *Drosophila *remodels the chromatin of the X chromosome in males to enhance the level of transcription of most X-linked genes, and thereby achieve dosage compensation. The core complex consists of five proteins and one of two non-coding RNAs. One of the proteins, MOF (males absent on the first), is a histone acetyltransferase that specifically acetylates histone H4 at lysine 16. Another protein, maleless (MLE), is an ATP-dependent helicase with the ability to unwind DNA/RNA or RNA/RNA substrates *in vitro*. Recently, we showed that the ATPase activity of MLE is sufficient for the hypertranscription of genes adjacent to a high-affinity site by MSL complexes located at that site. The helicase activity is required for the spreading of the complex to the hundreds of positions along the X chromosome, where it is normally found. In this study, to further understand the role of MLE in the function of the MSL complex, we analyzed its relationship to the other complex components by creating a series of deletions or mutations in its putative functional domains, and testing their effect on the distribution and function of the complex *in vivo*.

**Results:**

The presence of the RB2 RNA-binding domain is necessary for the association of the MSL3 protein with the other complex subunits. In its absence, the activity of the MOF subunit was compromised, and the complex failed to acetylate histone H4 at lysine 16. Deletion of the RB1 RNA-binding domain resulted in complexes that maintained substantial acetylation activity but failed to spread beyond the high-affinity sites. Flies bearing this mutation exhibited low levels of roX RNAs, indicating that these RNAs failed to associate with the proteins of the complex and were degraded, or that MLE contributes to their synthesis. Deletion of the glycine-rich C-terminal region, which contains a nuclear localization sequence, caused a substantial level of retention of the other MSL proteins in the cytoplasm. These data suggest that the MSL proteins assemble into complexes or subcomplexes before entering the nucleus.

**Conclusions:**

This study provides insights into the role that MLE plays in the function of the MSL complex through its association with roX RNAs and the other MSL subunits, and suggests a hypothesis to explain the role of MLE in the synthesis of these RNAs.

## Background

In *Drosophila*, dosage compensation (the equalization of many X-linked gene products between males with one X chromosome and females with two X chromosomes) is mediated by the male-specific lethal (MSL) complex, which consists of a core of five protein subunits (encoded by the genes *male-specific lethal (msl) 1*, *2 *and *3*, *males absent on the first *(*mof*), and *maleless *(*mle*)) and one of two non-coding RNAs (encoded by the genes *RNA on the X (rox) 1 and 2*). The complex preferentially associates with numerous sites on the X chromosome in somatic cells of males but not of females. It is responsible for an enhancement of the transcriptional rate of a large number of X-linked genes in males, thereby mediating a compensatory effect for the difference in dosage of these genes between males and females [1,2]. The presence of the MSL complex on the male X chromosome is correlated with the significant increase of a specific histone isoform: histone H4 acetylated at Lys16 (H4K16ac [3]). This acetylation is the result of the activity of MOF, a histone acetyltransferase of the MYST (MOZ, Ybf2/Sas3, Sas2, and TIP60) family [4-6]. In addition to this enzyme, the MSL complex of *Drosophila *includes an ATPase (MLE), a feature that distinguishes it from most of the other complexes that enhance transcription. MLE is an ATP-dependent DEXH-box RNA/DNA helicase that prefers double-stranded RNA or RNA/DNA hybrids [7].

In *Drosophila *females, the MSL complex does not assemble, because the product of the sex-regulatory gene *Sxl *prevents the translation of the *msl-2 *transcript [8,9]; the absence of MSL2, in turn greatly reduces the stability of MSL1 [10]. SXL is absent in males, and the complex is believed to assemble at the locus of the two *roX *genes and then spread to numerous additional sites along the X chromosome, for which it has a complete range of affinity levels [11,12]. Initially, approximately 40 of these sites were defined by immunofluorescence as 'high-affinity' sites because a partial complex that includes only MSL1 and MSL2 could bind to them [13]. A few individual sites were characterized without revealing unique similarity in sequence between them, or between them and the rest of the X chromosome [14-16], with the exception of two regions within the DNA hypersensitive sites associated with the *roX1 *and *roX2 *genes, which included several GAGA sequences [17]. Gilfillan *et al*. [18] identified some short sequences within clones that spanned a few high-affinity sites. These sequences shared the AGAGA motif or were generally A-rich, and the authors proposed that dispersed along the X chromosome are clusters of several distinct but degenerated sequence motifs, for which the MSL complex exhibits a complete range of affinities. Recently, Alekseyenko *et al*. [19] and Straub *et al*. [20] identified approximately 130 to 150 MSL complex-binding sites on the X chromosome with the common feature of GA dinucleotide repeats. From these sites that are used for spreading, wild-type complexes target activated genes [21,22].

The two *roX *RNAs are very different in size (the lengths of the predominant species are ~3.7 kb for *roX*1 and ~0.5 kb for *roX*2), yet can substitute for each other in the formation of fully functional MSL complexes [23]. Present in both RNAs, and required for their function, are a stem loop and three (*roX*1) or two (*roX*2) copies of a conserved sequence of eight nucleotides, the *roX *box [24,25]. The *roX *RNAs are unstable unless they associate with some of the MSL proteins, therefore it is reasonable to assume that the complex is assembled at the sites of transcription of the two *roX *genes [26]. Three of the complex proteins (MSL2, MSL3 and MOF) exhibit RNA-binding activity [27-29]. MLE can be dissociated from the complex bound along the X chromosome by treatment with RNase [30]. Following the discovery and identification of MOF as a member of the complex [4], this subunit and MSL3 were also found to be released by RNase treatment [27,31].

Three MLE mutations have been used extensively to analyze the role of this protein in the function of the MSL complex: *mle*^1^, which contains a stop codon truncating the protein after the first 125 amino acids; *mle*^ γ^^203^, a loss-of-function allele produced by an internal deletion [32], and *mle*^GET^, which encodes a protein with a single amino acid substitution (MLE (K413E) in the ATP-binding site, yielding an MLE protein with no ATPase and, therefore, no helicase activity [7]. In flies homozygous for the *mle*^γ^^203 ^mutant allele, the MSL1 and MSL2 proteins but not the MOF protein are found only at the high-affinity sites [33]. The presence of MLE (K413E) allows the formation of complexes that contain MSL1, MSL2, MSL3, MOF and the mutant MLE protein, but apparently, neither *roX*1 nor *roX*2 [34]. Recently, we showed that the ATPase activity of MLE is sufficient for the hypertranscription of genes that are adjacent to a high-affinity site by MSL complexes located at that site; the helicase activity is required for the spreading of the complex to the hundreds of positions at which it is normally found along the X chromosome [35].

At the onset of our experiments for this study, a number of functional interactions between the protein and RNA components of the MSL complex had been established. The MSL1 and MSL2 proteins form the X-chromosome-binding module of the complex [13], and the binding specificity of this module requires the association of MSL2 with *roX *RNA [29]. MSL1 also serves as a scaffold for the addition of MSL3 and MOF [36]. In *vitro*, association of MOF with the MSL1-MSL3 subcomplex results in a very substantial enhancement of the level and substrate specificity of its histone acetyltransferase activity [36]. In *vivo*, the addition of MSL3 and MOF to an assembling complex is normally dependent on the presence of *roX *RNA, but can be made to occur in the absence of this RNA by overexpression of MSL1 and MSL2 [37]. MLE is required for the incorporation of *roX *RNA in the MSL complex, and its own presence in the complex is mediated by this association [26,37]. Finally, the stem-loop region at the 3' end of *roX2 *RNA, and the *roX *box sequences at the 3' ends of *roX1 *and *roX2 *RNAs, are crucial for full histone acetylation by the MSL complex [24,25].

MLE has two putative RNA-binding domains, RB1 and RB2, in its N-terminal region, and a glycine-rich domain at its C-terminus. The function of these domains in RNA binding and in the targeting of the MSL complex to the X-chromosome territory has been studied *in vitro *and in S2 cultured cells [38]. These studies indicated that deletion of RB1 had no effect on the RNA-binding activity of MLE with respect to a double-stranded, 40 bp, substrate molecule with both strands flanked by single-stranded overhangs, or to a single-stranded substrate of approximately 60 bp, whereas deletion of RB2 eliminated binding to either type of RNA molecule. To better understand the role of MLE and of the *roX *RNAs in the targeting and spreading processes we created similar deletions in the two RNA-binding domains and in the glycine-rich region that includes the nuclear localization signal [38], and analyzed these proteins by assaying their ATPase and helicase activity and by determining their function in dosage compensation using a plasmid system [39]. We also determined the effect of mutant proteins on the assembly and function of the MSL complex by establishing transgenic fly lines that express them, and by monitoring the spreading and integrity of the MSL complex along the X chromosome in salivary-gland nuclei and the level of male viability of transgenic mutant males. In addition to using a different cell type, these experiments differ from those carried out with S2 cells by Izzo *et al*. [38], in that endogenous wild-type MLE was absent in the transgenic flies examined, and thus did not compete for inclusion in the MSL complex with the mutant MLE protein under study. Further, we used *in situ *hybridization on polytene chromosomes and quantitative reverse transcription (RT)-PCR to determine the cytological presence and nuclear levels of the *roX *RNAs.

Our experimental data provide new insights into the associations between the MSL subunits and *roX *RNA, which are a prerequisite for the function of this complex in dosage compensation.

## Results

**Figure 1:**
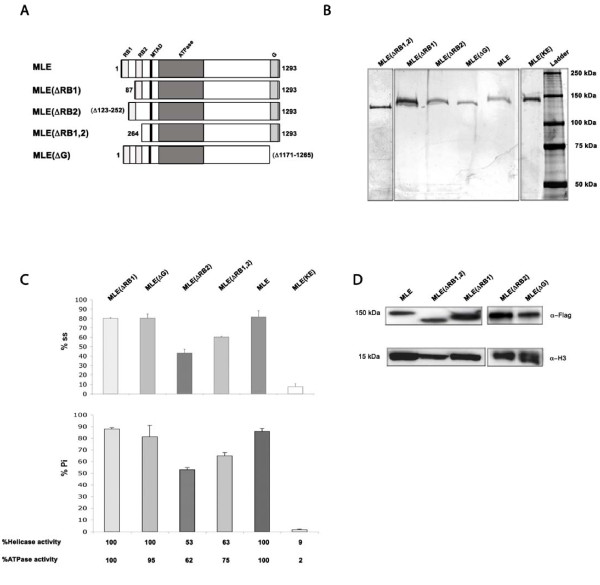
**Purification and assays of mutant maleless (MLE) proteins**. **(A) **Domain structure of the mutant MLE proteins. **(B) **Silver staining of purified Flag-tagged recombinant MLE proteins from S2 cells expressing the different transgenes run in a 7.5% SDS-polyacrylamide gel. **(C) **Results of ATPase and helicase assays of MLE proteins. Upper panel: helicase activity measured as a function of single-stranded RNA released from the double-stranded RNA/DNA substrate; lower panel: ATPase activity measured as a function of inorganic phosphate (Pi) released from radioactive ATP. The activity of wild-type MLE is set at 100%, and the error bars are the SD from the mean of three assays. **(D) **Expression of Flag-tagged wild-type and mutant MLE proteins in transgenic flies in a null endogenous *mle *gene background (*mle*^*1*^). Western blots of crude lysates from adult fly heads developed with anti-Flag antibodies (top panel), with anti-H3 antibodies as a loading control (bottom panel). Similar results were obtained with three independent lines of each transgene used for immunofluorescence and rescue experiments.

We constructed plasmids carrying cDNA sequences encoding N-terminal Flag-tagged MLE mutant proteins under the control of a metallothionein promoter, and deleted for each or both of the two double-stranded N-terminal RNA-binding motifs (RB1 and RB2) or the C-terminal glycine-rich domain (G) (Figure 1A). *Drosophila *S2 cells were transfected with these or similar plasmids to express the MLE(K413E) or wild-type MLE proteins (as negative and positive controls, respectively), and stable cell lines were established by hygromycin selection. Inexplicably, we were unable to establish a permanently transformed S2 cell line expressing MLE(ΔRB1), therefore we obtained the mutant protein from transiently transfected cells. Flag-tagged proteins were partially purified (Figure 1B), and tested for ATPase and helicase activities (Figure 1C). Proteins deleted for the first RNA-binding motif (amino acids 3-86: MLE(ΔRB1)) or for the glycine-rich motif (amino acids 1171-1265: MLE(ΔG)]) exhibit wild-type levels of ATPase and helicase activities. Proteins deleted for the second RNA-binding motif (amino acids 123-252: MLE(ΔRB2)) or for both motifs (amino acids 1-241: MLE(ΔRB1,2)) have reduced ATPase and therefore helicase activity.

We used our plasmid-based dosage-compensation model system [39] to determine if MSL complexes that include the different mutant MLE proteins are able to carry out dosage compensation. Control plasmids or plasmids bearing *roX *sequences (capable of dosage compensation) were transfected into stable S2 cell lines expressing the mutants. Although MLE(ΔRB2) exhibited substantial enhancement of gene activity, none of the mutant proteins tested supported full dosage compensation of the Firefly luciferase reporter gene (see Additional file 1).

To determine the effect of the various deleted MLE proteins on the distribution of the MSL complex and its function *in vivo*, transgenic lines carrying cDNAs encoding wild-type or mutant Flag-tagged MLE proteins were generated by germline transformation. Expression of the transgenes was determined by western blot analysis (Figure 1D). Each transgene was introduced into the genome of females carrying an *msl2 *cDNA insertion that allows the translation of its transcript; such females are able to assemble functional MSL complexes [8,9]. The presence of a homozygous null allele of the endogenous *mle *gene (*mle*^1^) causes the complexes to be incomplete and to bind only to a reduced number of sites [13]. Therefore, in female larvae of this genotype, the subunit constitution, the level of spreading beyond the high-affinity sites, and the extent of the H4K16 acetylation of the MSL complexes assembled with the Flag-MLE proteins can be determined by immunofluorescence on salivary-gland polytene chromosome spreads.

We also correlated the defects in MSL assembly and distribution of deleted MLE proteins with male viability by monitoring their ability to rescue *mle*^1 ^homozygous males. With one exception, a minimum of three independent transgenic lines was analyzed. None of the *mle *mutant transgenes was able to rescue *mle*^*1 *^homozygous males (Table 1). Western blot or silver-stained gel analysis indicated that the levels of the mutant MLE proteins purified from S2 cells for analysis of their ATPase and helicase activity were equivalent to the level produced by the wild-type transgene, and thus the mutations did not affect the stability of the proteins. Any problems related to the fact that the overexpression of transgenic gene products under the control of strong promoters can sometimes lead to spurious results were avoided in our rescue experiments by relying on the constitutive transcription of the uninduced hsp83 promoter for expression of the mutant transgenes.

**Table 1:** Rescue of *mle*^*1*^-induced male lethality by *mle *mutant transgenes.^a, b^

Transgene	**Females**^**c**^	**Males**^**c, d**^		
	+; *mle*^tg^	*mle*^1^, *mle*^tg^	+, *mle*^tg^	*mle*^1 ^; *mle*^tg^

*mle*^+ ^	138	84	139	79

*mle*(*ΔRB1*)	443	140	241	0

*mle*(*ΔRB2*)	486	160	421	0

*mle*(*ΔG*)	283	262	162	0

^a^Males and females without the transgene and heterozygous or homozygous for the *mle*^1 ^null allele are not included in the table.

^b^For each transgene, the progenies of three crosses using three independent transgenic lines are summed. The results with the *mle*^+ ^transgene are the sum of two independent transgenic lines.

^c^Progeny of the cross: *w/w *; *pr mle*^1^/*CyO *; *mle*^tg ^*w*^+^/+ x *w/*Y; *pr mle*^1^/*CyO *; *mle*^tg ^*w*^+^/+ or of the cross: *w/w *; *pr mle*^1^/*pr mle*^*1 *^; *mle*^tg ^*w*^+^/*mle*^tg ^*w*^+ ^x *w/*Y; *pr mle*^1^/*CyO *; *mle*^tg ^*w*^+^/+ in the case of *mle*(*ΔG*).

+; *mle*^tg ^: hemizygous or homozygous for the transgene.

^d^*mle*^1 ^; *mle*^tg ^: homozygous for the *mle*^1 ^null allele and hemizygous or homozygous for the transgene.

The results of the rescue experiments diverged from Punnett square expectations: females hemizygous or homozygous for the transgene should be twice as numerous as females homozygous for the *mle*^1 ^null allele and hemizygous or homozygous for the transgene, while males and females hemizygous or homozygous for the transgene should occur in equal numbers. With the exception of the progeny from the crosses involving an *mle*^+ ^transgene, the numbers of females homozygous for the *mle*^1 ^null allele and hemizygous or homozygous for the transgene were fewer than expected. This is consistent with the recent observation that the MSL complex is required in early female embryonic development for the upregulation of the female master regulatory gene *Sex-lethal*, and for the mechanism that counts the number of X chromosomes [40]. Mutant MLE proteins may interfere with formation of the complex, which normally results from the presence of maternal gene products (including the *msl2 *transcript). In males, in which the MSL complex is required in most tissues throughout life, the transgenes expressing a mutant MLE protein have a more pronounced dominant effect. These considerations notwithstanding, the transgenes expressing mutant MLE proteins clearly failed to rescue *mle*^1 ^mutant males.

### Deletion of RB1 allows spreading of an MSL complex that is largely deficient in MSL3, and fails to acetylate H4K16

**Figure 2:**
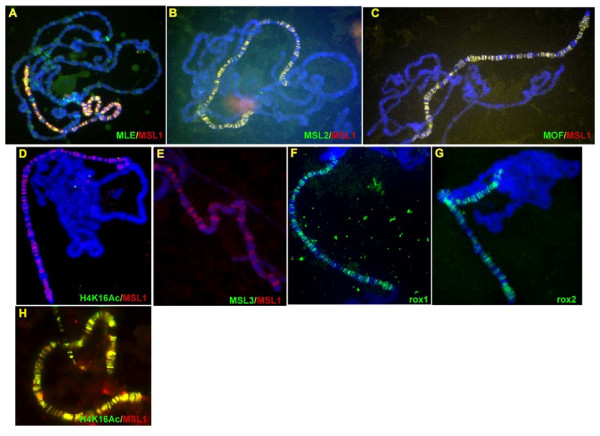
**Distribution of male-specific lethal (MSL) complex subunits on polytene chromosomes of larvae expressing a Flag-tagged maleless (MLE)(ΔRB1) protein**. **(A-E) **Indirect immunofluorescence was performed on polytene chromosomes from salivary glands of *y w*^*1118*^*/w; pr mle*^*1*^/*pr mle*^*1*^; *H83msl2/hsp83-Flag-mle(ΔRB1)w*^+ ^females. The only MLE epitope present was the product of the transgene. Preparations were stained with the two antisera indicated, and yellow signals an overlap of the respective epitopes. **(F, G) **Distribution of the *roX *RNAs in the presence of Flag-tagged MLE(ΔRB1) protein. *In situ *hybridization was performed on salivary-gland polytene chromosomes from females of the genotype indicated above. **(H) **Polytene chromosomes of a *w*^*1118*^*/w; pr mle*^*1*^/*pr mle*^*1*^; *H83msl2/hsp83-Flag-mle*^+^*w*^+ ^control female stained with anti-H4K16ac and anti-MSL1 sera. As expected, there was complete overlap of the two epitopes.

MSL complexes containing MLE(ΔRB1) were found throughout the polytenic X chromosomes. At this level of resolution, it was unclear whether this distribution is actually equivalent to that of the wild type. These mutant complexes included MSL1, MSL2 and MOF (Figure 2A-C), and *roX*1 and *roX*2 (Figure 2F-G), but neither the presence of MSL3 nor of H4K16ac was detectable (Figure 2D-E). The nuclear levels of the two *roX *RNAs were below the expected normal range of a fully compensated genotype, as determined by quantitative RT-PCR (see Additional file 2). It therefore appears that deletion of RB1 alters the interactions within the complex subunits, including the roX RNA, in a manner that prevents the stable association of MSL3 [31]. In turn, the lack of acetylation of H4K16 may be explained by the observation that *in vitro*, the activity of MOF is dependent on its association with MSL1 and MSL3 [36].

### Deletion of RB2 causes a differential distribution of the MSL subunits

**Figure 3:**
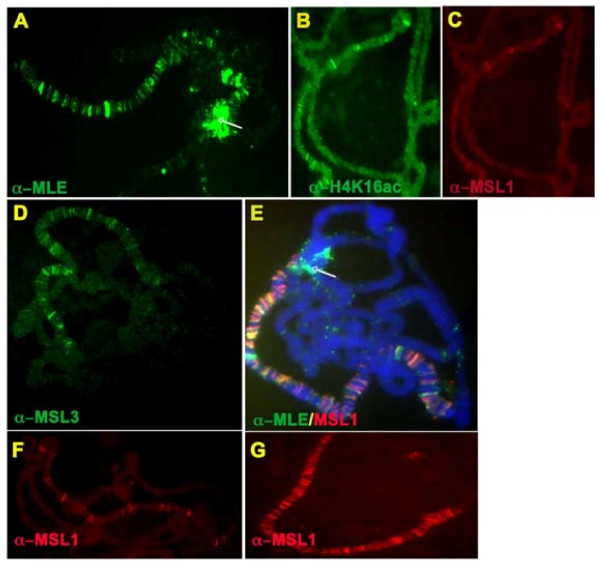
**Distribution of male-specific lethal (MSL) complex subunits on polytene chromosomes of larvae expressing a Flag-tagged maleless (MLE)(ΔRB2) protein**. Indirect immunofluorescence was performed on polytene chromosomes from salivary glands of *y w*^*1118*^*/w; pr mle*^*1*^/*pr mle*^*1*^; *H83msl2/hsp83-Flag-mle(ΔRB2)w*^+ ^females. **(B-D) **MSL1, MSL3 and H4K16ac were restricted to the high-affinity sites, whereas **(A, E) **MLE was associated with additional sites and was present prominently in the chromocenter (white arrows). Similar results (data not shown) were obtained on salivary-gland polytene chromosomes from females expressing the *hsp83-Flag-mle(ΔRB1,2)w*^+ ^transgene. **(F, G) **Control females homozygous for (F) H83 *msl2 *and *mle*^*1 *^(G) H83 *msl2 *and *Flag-mle*^*+*^. For the wild-type distribution of H4K16ac please see Figure 2H.

Fully formed complexes consisting of MLE(ΔRB2) (Figure 3B-D) or MLE(ΔRB1,2) (data not shown) and the other MSL subunits were found only at a limited number of sites, which we identified as the high-affinity sites on the basis of the cytological map location of a selected subset [13]. In most nuclei, the deleted MLE proteins appeared to associate independently with additional sites (Figure 3E), and were also found at the chromocenter (Figure 3A, E). In the case of both mutant MLE proteins, the two *roX *RNAs were not detectable by *in situ *hybridization, and their nuclear levels, as determined by quantitative RT-PCR, were greatly reduced (see Additional file 2). The deficiency in the level of these RNAs may be responsible for the ectopic binding of the mutant MLE proteins. Acetylated H4K16 was present at the entry sites in the polytene chromosomes of transgenic larvae expressing MLE(ΔRB2) but not MLE(ΔRB1,2). As expected, there was no rescue of *mle*-null males (Table 1). Low levels of *roX *RNA may be sufficient for the assembly of a small number of complexes that are limited to the high-affinity sites, explaining the H4K16 acetylation at these sites.

### Deletion of the glycine-rich C-terminal domain leads to a substantial level of cytoplasmic sequestration of the MSL protein subunits, and a disorganization of the complex within the nuclei

**Figure 4:**
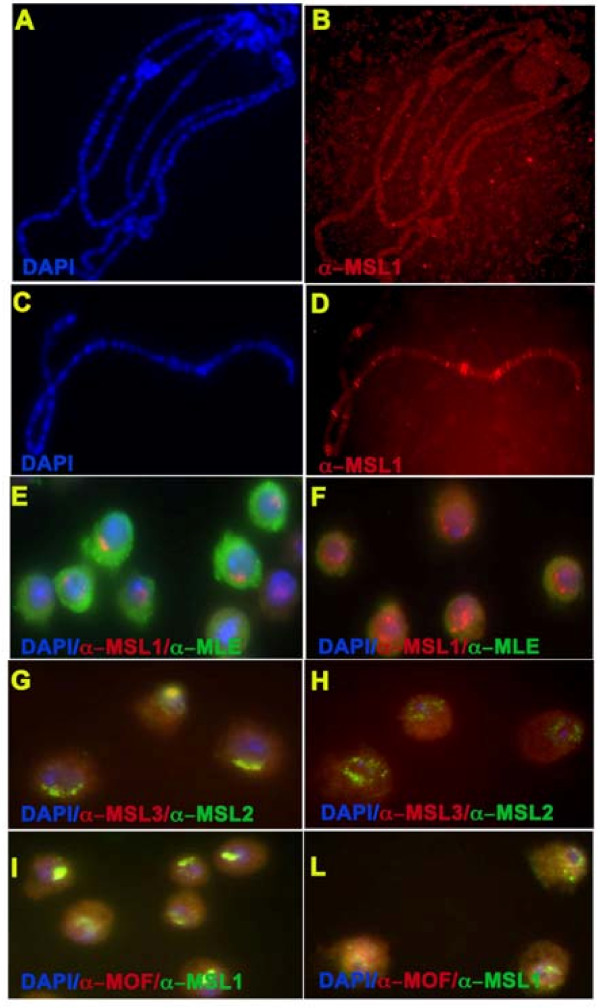
**Distribution of male-specific lethal (MSL) proteins in the presence of Flag-tagged maleless (MLE)(ΔG) protein**. Indirect immunofluorescence was performed on salivary-gland polytene chromosomes from **(A, B) ***y w*^*1118*^*/w; pr mle*^*1*^/*pr mle*^*1*^; *H83msl2/hsp83-Flag-mle(ΔG)w*^+ ^females and **(C, D) **control *y w*^*1118*^*/w; pr mle*^*1*^/*pr mle*^*1*^; *H83msl2/H83msl2 *females. Although MSL1 was present as expected at the high-affinity sites in the control females, it seemed to be completely absent in the females expressing the *Flag-mle(ΔG)w*^+ ^transgene. Indirect immunofluorescence was performed on S2 cells overproducing the Flag-MLE(ΔG) protein and treated with either **(E, G, I) **green fluorescent protein (GFP)-tagged double-stranded (ds)RNA or **(F, H, L) **dsRNA complementary to the region encoding the amino acids in the ΔG deletion **(E, F)**. The presence of endogenous wild-type MLE in the GFP dsRNA-treated cells resulted in the presence of MSL complexes in the X chromosome nuclear compartment, while the Flag-MLE(ΔG) deficient in the nuclear localization sequence remained in the cytoplasm. In the absence of wild-type MLE, a substantial amount of **(F) **MSL1, **(H) **MSL2 and MSL3 and **(L) **MOF (males absent on the first) remained in the cytoplasm. In these cells, MSL1, MSL2 and MOF were present in the nuclei, but were not confined to the X-chromosome compartment. For the wild-type distribution of H4K16ac please see Figure 2H.

In polytene chromosome spreads from salivary glands of transgenic females expressing MLE(ΔG), there was not visible association of MSL1, MSL2, MOF or MLE with the X chromosomes (Figure 4A, B). This is in contrast to control females carrying the MSL2 transgene but in which MLE was absent due to the presence of *mle*^1 ^mutant alleles: in these females, the remaining MSLs were present at the high-affinity sites (Figure 4C, D, and data not shown). Therefore, although the absence of MLE in MLE(ΔG) transgenic lines can be explained by the fact that the deletion includes a significant portion of the nuclear localization sequence [38], the lack of association of the other subunits, especially MSL1 and MSL2, with the high-affinity sites was surprising. To determine if these proteins were present in the nucleus, we examined the cells of the stable S2 line that overproduces Flag-MLE(ΔG) and in which expression of the endogenous *mle*^+ ^gene was knocked down by RNA interference targeted to the MLE sequence included in the ΔG deletion (see Additional file 1), and in control cells treated with green fluorescent protein double-stranded RNA. The presence of endogenous wild-type MLE in the latter cells resulted in the occurrence of MSL complexes in the X-chromosome nuclear compartment, while the Flag-MLE(ΔG) deficient in the nuclear localization sequence (NLS) remained in the cytoplasm (Figure 4E, F). In the absence of wild-type MLE, a substantial amount of MSL1 (Figure 4F), MSL2 and MSL3 (Figure 4H) and MOF (Figure 4L) also remained in the cytoplasm. In these cells, MSL1, MSL2 and MOF were present in the nuclei but were not confined to the X-chromosome compartment. As expected, the levels of the two *roX *RNAs, as determined by RT-PCR, were also extremely low (data not shown).

## Discussion

### Functional interactions between the MSL protein subunits

In transgenic females expressing a translatable *msl2 *transcript and the MLE(ΔRB1) protein (which is unable to bind *roX *RNAs), complexes containing four MSL proteins and *roX *RNAs were found to be spread along the X chromosomes. This observation suggests that the *roX *RNA-binding activity of MLE is not crucial for the incorporation of these RNAs in the MSL complex. We found that the level of association of MSL3 with the other MSLs in these complexes was greatly reduced. Although a deletion of MSL3 is known to prevent the complex from spreading along the X chromosome, it is possible that the presence of a few complexes that do contain MSL3 enables the spreading of the MSL3-depleted forms. The complete absence of any H4K16ac signal was unexpected, and indicates that an interaction between MLE and another component of the complex required for MOF activity was disrupted by the RB1 deletion. Li *et al*. [[29] reported that the amino-terminal region of MSL2 is sufficient to impart MOF activity and H4K16 acetylation capability to complexes, albeit mislocalized to the chromocenter. Further, these authors were able to co-immunoprecipitate only MLE with a C-terminal fragment of MSL2. They proposed that the proline-rich motif in MSL2 (amino acids 685 to 713) combines with one of the RNA-binding domains of MLE to form a module that specifically binds roX RNAs. Consistent with these observations, our results suggest that the MLE(ΔRB1) protein interacts with MSL2 in a manner that prevents MOF activation, either by inducing an inappropriate conformational change or by preventing its normal, mutual association with roX RNA.

The evidence of a functional interaction between MOF and MLE-MSL2, and between MOF and MSL3 as mentioned above, suggests an explanation for the observation that a single amino acid substitution [*mof*^1^, MOF(G691E)], leading to loss of function in MOF, prevents a fully assembled complex from spreading beyond the entry sites [33]. In all probability, the lack of H4K16 acetylation is not responsible for the lack of spreading; rather, because the *mof*^1 ^mutation occurs in the putative acetyl co-enzyme A binding site, failure of MOF to bind the co-enzyme may not only result in the expected loss of acetylation function, but also in a conformational change that, through interactions with MLE-MSL2, directly or indirectly affects the abilities of MLE, for example, to achieve spreading of the complex.

**Figure 5:**
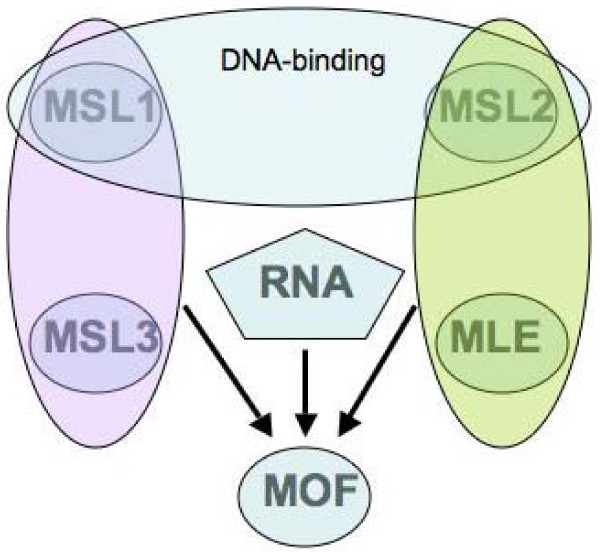
**Summary of the known interactions of the male-specific lethal (MSL) complex subunits**. MSL1 and MSL2 form a DNA-binding module, the specificity of which requires the association of MSL2 with roX RNA. The association of MSL1 and MSL3 with MOF enhances its histone acetyltransferase activity and specificity. MLE and MSL2 interact and contribute to MOF activity. Maleless (MLE) is responsible for the incorporation of roX RNA in the complex and requires roX RNA to associate with the complex. The roX RNA is needed for full histone acetylation. Please see the discussion section for details of these associations.

Deletion of the second RB motif leads to a protein that partially lacks ATPase and helicase activities. This level of enzymatic function is insufficient to allow the spreading of the MSL complex beyond the high-affinity sites [35]. We found that H4K16ac was present at these sites in MLE(ΔRB2)-bearing transgenic females but not in MLE(ΔRB1,2) females, consistent with the role of RB1 on MOF activity *in vivo*. Extending the argument made in the preceding paragraph, only the RB1 sequence (deleted in MLE(ΔRB1)) interacted with MSL2 to enable MOF function, whereas the RB2 sequence deleted in MLE(ΔRB2) did not and the acetyl transferase activity of MOF was maintained. A summary of the known interactions of the MSL complex subunits is presented in Figure 5.

### Functional interactions of the MSL proteins with roX RNAs

Deletion of the second RNA-binding domain resulted in greatly reduced nuclear levels of *roX *RNAs. This observation can be explained by the possibility that MLE contributes to the stability or perhaps to the synthesis of roX RNA. The RB2 site is necessary for the incorporation of the roX RNAs into the complex and, in absence of their association with the complex proteins, these RNAs are degraded. An alternative possibility is that the RB2 site is responsible for the transcriptional regulation of the *roX *genes, as described by Lee *et al*. [41]. This latter conclusion suggests a possible mechanistic basis for the known effect of MLE on *roX *gene transcription [42]. DEAD-box helicases are often present at transcription sites, where they are thought to facilitate co-transcriptional processes such as 5' end capping and intron splicing [43,44]. An additional function may be to prevent or diminish the association of nascent RNA with DNA at the site of transcription, leading to the formation of DNA-RNA hybrids that can interfere with subsequent rounds of transcription [45]. The *in vitro *preference of MLE for unwinding DNA-RNA hybrid substrates with single-stranded RNA regions [7] would be concordant with such a function. The MLE(ΔRB2) protein, lacking the ability to bind RNA, would be incapable of resolving DNA-RNA hybrids resulting from transcription, leading to reduced *roX *RNA levels.

### Do the MSL proteins interact in the cytoplasm before their entry into the nucleus?

In polytene chromosome spreads of transgenic females expressing the MLE(ΔG) protein and a translatable *msl2 *transcript, MSL1, MSL2, MOF and mutant MLE were absent from the X chromosome. The levels of the two *roX *RNAs were extremely low, although they were somewhat higher than in wild-type females, perhaps because of a difference in the genetic background in our experiments. The deletion included a portion of the nuclear localization sequence, and MLE(ΔG) was completely absent from the nucleus in whole salivary-gland nuclei of transgenic females and in the nuclei of S2 cells, where the endogenous MLE was knocked down by RNA interference. All of the other MSL proteins were found at substantial levels in the cytoplasm. This observation was unexpected, because in females expressing the translatable *msl2 *transcript and that were homozygous for the *mle*^1 ^null mutation, MSL1 and MSL2 were present at the high-affinity sites. A possible explanation is that the presence of MLE(ΔG) exclusively in the cytoplasm sequesters the other MSL proteins, perhaps by forcing them to interact in this compartment. This raises the possibility that in wild-type male cells, some of the MSL proteins may assemble into complexes or subcomplexes before entering the nucleus, and that this cytoplasmic assembly does not require *roX *RNA. In the absence of MLE and MSL2 in the nucleus, the transcription of the *roX *genes is then not induced [46,47], and this may explain the absence of properly targeted MSL proteins in the nucleus that we saw in our study.

To date, a large number of complexes that modify or remodel chromatin have been identified and their subunits described. Understanding the interaction of the subunits of a particular complex is often limited to determining whether its function is allowed or abrogated by deletion of one or more subunits. The MSL complex offers the advantage that its core is made up of only five proteins and an RNA component, facilitating the task of describing in greater detail the associations of these components and the role that these associations play in the assembly, targeting and function of the complex. Clearly, the next level of understanding should be sought with biophysical approaches for which the observations described in this paper and those of others will provide valuable guidance.

## Conclusions

We have characterized the role that RNA binding plays in the association of the MSL complex with MLE, and the function of the MSL complex. We have provided *in vivo *evidence that MLE may contribute to the synthesis of the roX RNAs, and that some of the proteins of the complex may associate with one another before their entry into the nucleus.

## Methods

### Plasmid construction

All recombinant clones were constructed using the cloning strategies described previously [35]. The ΔRB1 (amino acids 3-86), ΔRB2 (amino acids 123-252) and ΔG (amino acids 1171-1265) deletions were initially made by digesting out the regions of interest from the full length Flag-MLE/pBS construct (full length N-terminal Flag-tagged MLE cDNA in pBlueScript vector), and replacing them with a linker containing the compatible overhangs and desired amino acids between the two internal restriction sites. All subclones were verified by sequencing. For construct ΔRB1,2 (amino acids 1-241), a combination of PCR (performed with appropriate sets of primers) and restriction enzyme digestion were used. The generated Flag-tagged mutants were subcloned in both pMK33 and pCasperhs83 vectors as previously described [35]. All vectors and primers used are listed in Additional file 3.

### Transfections and selection of stable lines

*Drosophila *S2 cells were transfected with each recombinant *mle*-pMK33/pMtHy vector using a transfection reagent (Effectene; Qiagen Inc., Valencia, CA, USA). Stably transfected cells were selected with increased amounts of hygromycin B (Cellgro; Mediatech Inc., Manassas, VA, USA). Stably transfected cells grown to 3 × 10^6 ^cells/ml were transferred to 500-ml spinner flasks and cultured at 25°C with constant stirring (80 rpm) until a doubling of the cell density with a viability of >90% was reached. Copper sulfate (200 μmol/l) was added to induce production of recombinant MLE protein, and the flasks incubated for 24 h.

### Purification of the Flag-tagged MLE recombinant proteins

Preparation of nuclear extracts was performed as described previously [6], using a salt-extraction protocol. Extracts were mixed with anti-Flag M2-agarose beads (Sigma Chemical Co., St Louis, MO, USA) equilibrated with nucleus extraction buffer with high salt (350 mmol/l NaCl), gently rocked at 4°C for 1 h, and then loaded onto a 5-ml column. The beads were washed with five volumes of nucleus extraction buffer with 350 mM NaCl, followed by five volumes of low-salt extraction buffer (150 mmol/l NaCl). Bound Flag-MLE proteins were eluted with 200 μg/ml of Flag peptide and 20% glycerol in low-salt extraction buffer. Aliquots were quickly frozen in liquid nitrogen and kept at -80°C. The purity of each protein preparation was checked by 7.5% sodium dodecyl sulfate (SDS)-polyacrylamide gel electrophoresis, using a silver-staining protocol.

### Helicase and ATPase assays

ATP-dependent RNA helicase activity was measured as described previously [7], using the same dsRNA substrate and 5 ng of each Flag-tagged recombinant MLE protein per assay. ATPase activity was measured as described previously [48] at pH 7.6 in 20-μl reaction mixtures containing 5 ng of each Flag-tagged recombinant MLE protein per assay. The reaction mixtures were spotted onto a polyethyleneimine thin-layer chromatography plate (Sigma Chemical Co.). ATP and P_i _were separated by chromatography in 1 mol/l formic acid/0.5 mol/l LiCl for 45 min and then located by autoradiography. All enzyme reaction products were quantified on a phosphorimager.

### Transgenic lines

Flag-tagged recombinant *mle*/pCasperhs83 constructs were purified (Qiaprep Spin Miniprep Kit; Qiagen), and used for germline transformation of a *w1118 *mutant strain (Genetic Services, Inc., Cambridge, MA, USA). The G_1 _progeny were mated with *w1118*; *T*(*2*;*3*)*apXa/CyO P*{*ActGFP.w*^-^}*CC2*;*TM6 Sb Tb *flies to determine the chromosome of insertion. Males, *w1118/Y*; *pr mle1/Bc*; [*hsp83-Flag-mle*^tg ^w^+^]*/TM6 Sb Tb *(where *mle*^tg ^indicates a transgene), were then mated with *y w*; *pr mle1/pr mle1*; *H83msl2/H83msl2 *females to monitor the rescue of homozygous *mle1 *male lethality, and to visualize the localization of MSL complexes carrying the Flag-MLE recombinant proteins on female polytene chromosomes.

### Cytoimmunofluorescence

Glands were dissected from third-instar *y w*^*1118*^*/w*^*1118*^; *pr mle*^*1*^*/pr mle*^*1*^; *H83msl2/hsp83-Flag-mle*^tg ^*w*^+ ^female larvae, and polytene chromosomes were prepared for immunofluorescence as previously described [21]. After 24 h of induction with copper, 1 × 10^6 ^S2 cells/ml were seeded onto slides and allowed to settle for 1 h. Subsequently, cells were washed in phosphate-buffered saline (PBS) and fixed with 4% paraformaldehyde in PBS for 15 minutes. After permeabilization with 0.5% Triton-X for 5 minutes, cells were washed four times for 5 minutes each in PBS. Slides were blocked with 12% normal donkey serum (Jackson Immunoresearch Laboratories, Inc., West Grove, PA, USA) and 0.2% Tween in PBS for 30 minutes. Cells were incubated with antibody for 1 hour at room temperature. After washing in PBS, slides were stained for 1 hour with Cy3- and fluorescein isothiocyanate-labeled secondary antibodies (Jackson Laboratories, Inc.) diluted in blocking buffer. Slides were mounted using medium (Vectashield, Vector Laboratories Inc., Burlingame, CA, USA) containing 4',6-diamidino-2-phenylindole (DAPI).

### Quantitative RT-PCR of *roX *RNAs

Total RNA was extracted from third-instar larvae (Trizol Plus RNA Purification Kit; Invitrogen) and subsequently treated with DNase (Turbo DNase; Ambion Inc., Austin, TX, USA). Quantitative RT-PCR was performed in triplicate on at least three different preparations with the roX1 and roX2-specific primer pairs (see Additional file 3) using a thermal cycler (iCycler; Bio-Rad Laboratories, Inc., Hercules, CA, USA). The data were analyzed as follows: fold difference is the ratio of 2^ΔCt (control minus target gene product in mutant sample) divided by 2^ΔCt (control minus target gene product in wild-type sample). Rp49 RNA was used as control to normalize the data.

### *In situ *hybridization

Fluorescence *in situ *hybridizations of polytene chromosome were performed as previously described [49]. Briefly, single-stranded antisense *roX1 *and *roX*2 probes were labeled by *in vitro *transcription with a biotin RNA labeling mix (RocheApplied Science, Indianapolis, IN, USA) with 1.6 kb *roX1 *(nucleotides 1536 to 3110) or 0.8 kb *roX2 *cDNA fragments in pTopo 2.1 vector as the template. Chromosome spreads were fixed in 4% formaldehyde, treated with proteinase K, washed in glycine, and prehybridized at 42°C for 3 h. Hybridizations containing biotinylated riboprobe were carried out overnight at 42°C. The avidin-biotin system (Vector Laboratories) was used for detecting hybridized biotin-labeled probes under conditions recommended by the manufacturer. Slides were mounted in mounting medium (Vectashield; Vector Laboratories) containing DAPI.

## Competing interests

The authors declare that they have no competing interests.

## Authors' contributions

RM carried out most of the experimental work. RY contributed to the cloning and the synthesis of transgenes. HL contributed to the experiments with the plasmid system and *in situ *hybridization. RM and JCL were responsible for the study design and manuscript preparation. All authors read and approved the final manuscript.

## Supplementary Material

Additional file 1 **Supplementary figure 1**. Left panel: levels of dosage compensation in S2 cells forming male-specific lethal (MSL) complexes that include the different mutant maleless (MLE) proteins. *roX *sequence-bearing plasmids (X) capable of dosage compensation, or control plasmids (N), were transfected into 'wild-type' S2 cells or into stable S2 cell lines expressing the mutants [39]. None of the mutant MLE proteins tested supported full dosage compensation of the Firefly luciferase reporter gene (expressed as the relative ratio of the Firefly luciferase gene in *roX*-bearing and control plasmids). Right panel: Double-stranded (ds)RNA complementary to the sequence encoding the amino acids in the deletion of the Flag-MLE(ΔG) protein abolished wild-type MLE produced by the endogenous gene of S2 cells. Thus, Flag-MLE(ΔG) mutant cannot support dosage compensation of the reporter gene. Click here for file

Additional file 2 **Supplementary figure 2**. Nuclear levels of *roX1 *and *roX2 *RNAs were determined by quantitative reverse-transcriptase PCR in transgenic larvae. The RNA was isolated from control y *w*^*1118*^*/w; pr mle*^*1*^*/pr mle*^*1*^; H83*msl2*/H83*msl2 *females and males (lanes 1 and 2, respectively) and *y w*^*1118*^*/w; pr mle*^*1*^*/pr mle*^*1*^; H83*msl2*/*hsp83-Flag-(mle*^*tg*^*)w+ *transgenic larvae (lanes 3 and 4). For reference purposes, roX RNA levels were determined in Oregon-R wild-type males and females (lanes 5 and 6, respectively). The absence of wild-type *mle *alleles led to a significant reduction in the synthesis of the two roX RNAs. The presence in the genome of the *mle*(ΔRB1) or *mle*(ΔRB2) transgenes did not further affect the level of these RNAs. H83*msl2 *is a transgene that expresses the MSL2 protein under the control of the hsp83 promoter and allows females to assemble a male-specific lethal (MSL) complex. Click here for file

Additional file 3 **Supplementary methods**. Supplementary methods for dosage compensation assay, reverse-transcriptase-PCR of roX RNAs and plasmid construction. Click here for file
