# Supplementary material for: Role of the ATPase/helicase maleless (MLE) in the assembly, targeting, spreading and function of the male-specific lethal (MSL) complex of Drosophila
Source: Epigenetics Chromatin. 2011 Apr 12;4:6. doi: 10.1186/1756-8935-4-6 (PMC3096584; doi:10.1186/1756-8935-4-6)

**Additional file 1 Supplementary figure 1.** Left panel: levels of dosage compensation in S2 cells forming MSL complexes that include the different mutant MLE proteins**.** *roX* sequence-bearing plasmids (X) capable of dosage compensation or control plasmids (N) were transfected into “wild type” S2 cells or into stable S2 cell lines expressing the mutants [39]. None of the mutant MLE proteins tested supported full dosage compensation of the Firefly luciferase reporter gene (expressed as the relative ratio of the Firefly luciferase gene in *roX*-bearing and control plasmids). Right panel: dsRNA complementary to the sequence encoding the amino acids in the deletion of the Flag-MLE(∆G) protein abolish the presence of wild type MLE produced by the endogenous gene of S2 cells. Thus, Flag-MLE(∆G) mutant cannot support dosage compensation of the reporter gene.


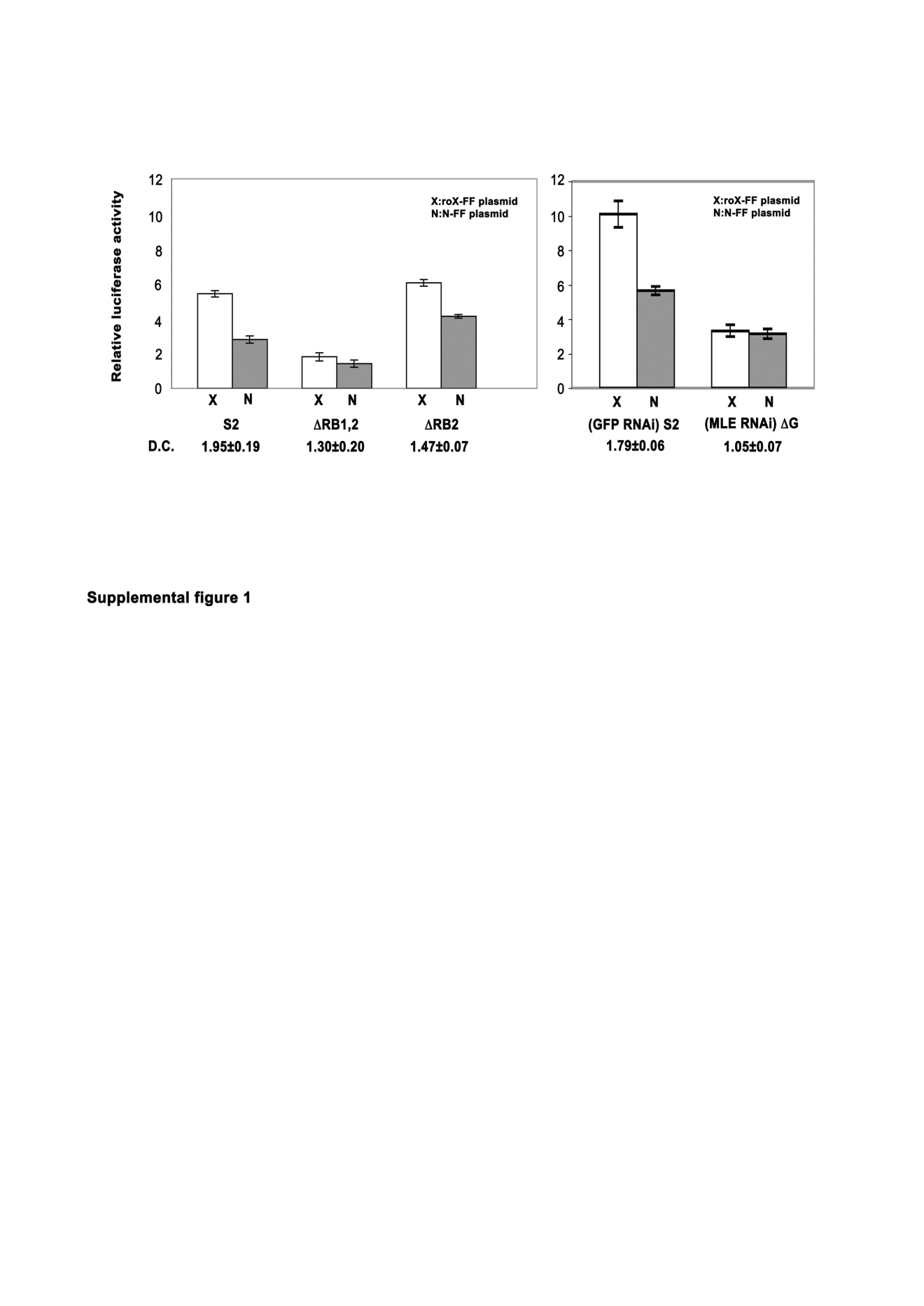

Supplement: Additional file 1 — Supplementary figure 1. Left panel: levels of dosage compensation in S2 cells forming male-specific lethal (MSL) complexes that include the different mutant maleless (MLE) proteins. roX sequence-bearing plasmids (X) capable of dosage compensation, or control plasmids (N), were transfected into 'wild-type' S2 cells or into stable S2 cell lines expressing the mutants [39]. None of the mutant MLE proteins tested supported full dosage compensation of the Firefly luciferase reporter gene (expressed as the relative ratio of the Firefly luciferase gene in roX-bearing and control plasmids). Right panel: Double-stranded (ds)RNA complementary to the sequence encoding the amino acids in the deletion of the Flag-MLE(ΔG) protein abolished wild-type MLE produced by the endogenous gene of S2 cells. Thus, Flag-MLE(ΔG) mutant cannot support dosage compensation of the reporter gene. [file 1756-8935-4-6-S1.DOCX]
