# Supplementary material for: Role of the ATPase/helicase maleless (MLE) in the assembly, targeting, spreading and function of the male-specific lethal (MSL) complex of Drosophila
Source: Epigenetics Chromatin. 2011 Apr 12;4:6. doi: 10.1186/1756-8935-4-6 (PMC3096584; doi:10.1186/1756-8935-4-6)

**Additional file 2 Supplementary figure 2.** Nuclear levels of *roX1* and *roX2* RNAs were determined by quantitative RT-PCR in transgenic larvae. The RNA was isolated from control y *w^1118^/w; pr mle^1^/pr mle^1^*; H83*msl2*/H83*msl2* female and male (lanes 1 and 2) , and *y w^1118^/w; pr mle^1^/pr mle^1^*; H83*msl2*/*hsp83-Flag-(mle^tg^)w+* transgenic larvae (lanes 3 and 4). For reference purposes, roX RNA levels were determined in Oregon-R wild type males and females (lanes 5 and 6). The absence of wild type *mle* alleles leads to a significant reduction in the synthesis of the two roX RNAs. The presence in the genome of the *mle*(ΔRB1) or *mle*(ΔRB2) transgenes does not further affect the level of these RNAs. H83*msl2* is a transgene that expresses the MSL2 protein under the control of the hsp83 promoter and allows females to assemble an MSL complex.


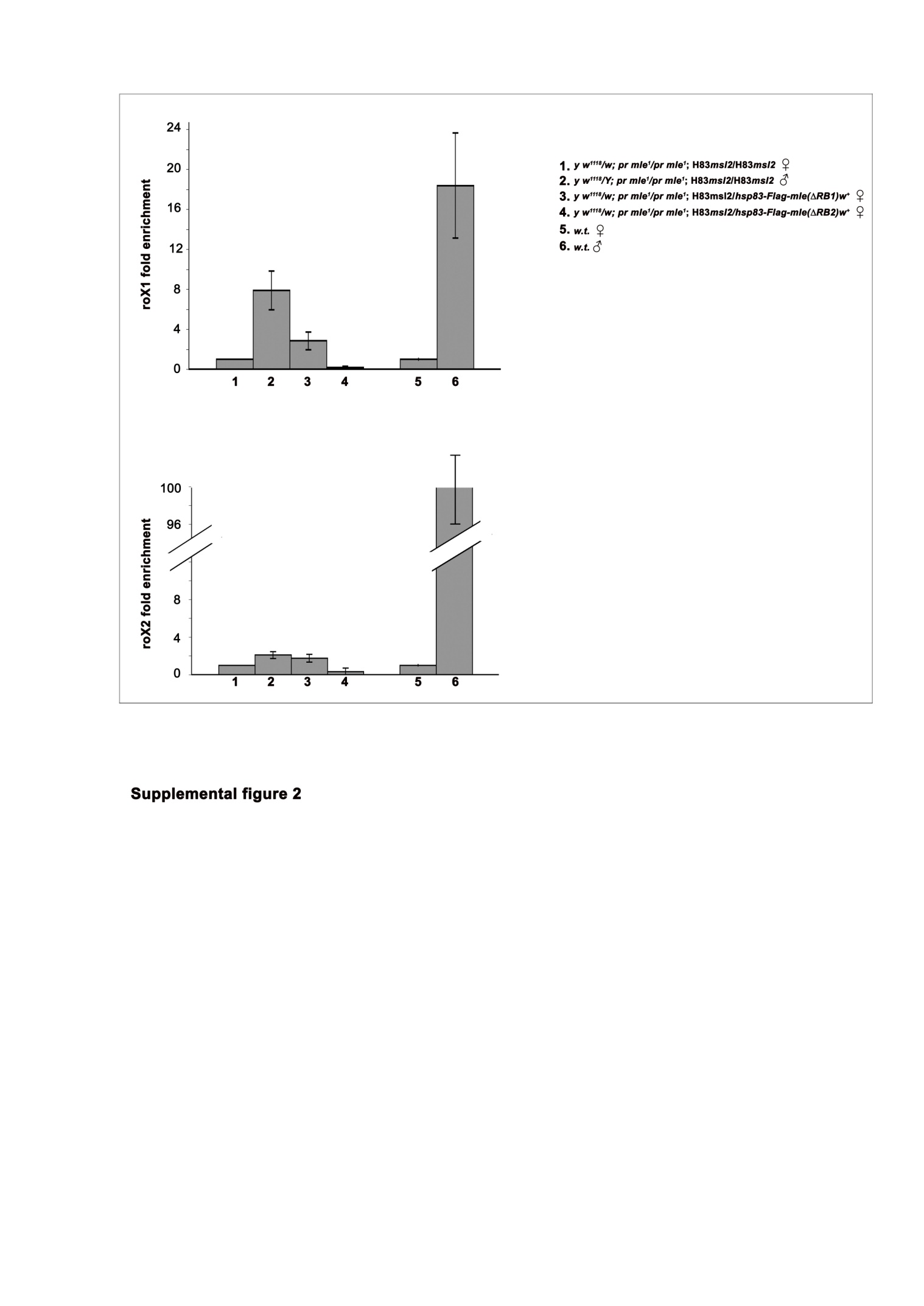

Supplement: Additional file 2 — Supplementary figure 2. Nuclear levels of roX1 and roX2 RNAs were determined by quantitative reverse-transcriptase PCR in transgenic larvae. The RNA was isolated from control y w1118/w; pr mle1/pr mle1; H83msl2/H83msl2 females and males (lanes 1 and 2, respectively) and y w1118/w; pr mle1/pr mle1; H83msl2/hsp83-Flag-(mletg)w+ transgenic larvae (lanes 3 and 4). For reference purposes, roX RNA levels were determined in Oregon-R wild-type males and females (lanes 5 and 6, respectively). The absence of wild-type mle alleles led to a significant reduction in the synthesis of the two roX RNAs. The presence in the genome of the mle(ΔRB1) or mle(ΔRB2) transgenes did not further affect the level of these RNAs. H83msl2 is a transgene that expresses the MSL2 protein under the control of the hsp83 promoter and allows females to assemble a male-specific lethal (MSL) complex. [file 1756-8935-4-6-S2.DOCX]
