# Supplementary material for: Role of the ATPase/helicase maleless (MLE) in the assembly, targeting, spreading and function of the male-specific lethal (MSL) complex of Drosophila
Source: Epigenetics Chromatin. 2011 Apr 12;4:6. doi: 10.1186/1756-8935-4-6 (PMC3096584; doi:10.1186/1756-8935-4-6)
Supplement: Additional file 3 — Supplementary methods. Supplementary methods for dosage compensation assay, reverse-transcriptase-PCR of roX RNAs and plasmid construction. [file 1756-8935-4-6-S3.DOCX]

**Additional file 3 Supplementary methods.** File includes supplementary methods for dosage compensation assay, RT-PCR of roX RNAs and plasmid construction.

**Dosage compensation assay of complexes containing mutant MLE proteins.** If normal dosage compensation is operating on the *roX* sequence-bearing plasmid, the relative level of Firefly luciferase is expected to be twice that of the control plasmid. Expression of the Flag-MLE(∆RB1,2) or the Flag-tagged MLE(∆RB2) mutant proteins substantially reduces the function of the MSL complex. The Flag-MLE(∆G) protein is unable to translocate to the nucleus and the cells that are transfected with this protein exhibit normal levels of dosage compensation due to the exclusive presence of wild type MLE in the nucleus. When this MSL subunit is eliminated by RNA interference, no dosage compensation can occur.

The plasmid model of dosage compensation [39] was used as previously described [35]. The Flag-MLE stable S2 lines were split to 30 to 60% confluence prior to transfection, which was carried out following the Qiagen Effectene protocol with 15 ng of supercoiled *roX*-bearing firefly luciferase plasmid (roX-FF) or Nesprin-bearing firefly luciferase (N-FF) plasmid (where Nesprin is 1 kb of the human *Nesprin* intron), 1 ng tTA activator plasmid, 5.4 ng of *Renilla* luciferase plasmid, and 1.2 μg of pBluescript (Stratagene) carrier; 10.8 μl of enhancer and 19.2 μl of Effectene reagent were used per 5 × 10^6^ cells. Just prior to transfection, 200 μM of copper sulfate was added to the medium. The next day, the cells were split to a final concentration of 0.3 × 10^6^ cells/ml, maintaining the same copper sulfate concentration. Three to five days after transfection, the cells were collected for luciferase assay and protein isolation. Luciferase activity was determined using the Dual Luciferase Reporter Assay system (Promega). The firefly luciferase activity was normalized to *Renilla* luciferase activity for each sample. At least three independent experiments were performed.

**RNA levels determined by real-time RT-PCR**.

Total RNA was extracted from 12 third-instar larvae using the Trizol Plus RNA purification kit (Invitrogen) and subsequently treated with Turbo DNase (Ambion). Quantitative reverse transcription (RT)-PCR was performed in triplicate on at least three different preparations with the respective gene-specific primer pairs using a Bio-Rad iCycler:

roX1 (Forward): 5’GAGAAGATTATACTAACCAGTTTGC-3’

(Reverse): 5’-AAGGAACAATTAAGAACAATTGAC – 3’

roX2 (Forward): 5’-GTCTTACGGACAGTGAGATGAG – 3’

(Reverse): 5’- CTTCAGTTTGCATTGCGACTT – 3’

RP49 (Forward): 5ʼ- AGAGTTCTTGTAACGTGGTCGGAATA-3ʼ

(Reverse): 5ʼ- CAATGGTGCTGCTATCCCAATC-3ʼ

The data were analyzed as follows: fold difference is the ratio of 2^∆Ct (control minus target gene product in mutant sample) / 2^∆Ct (control minus target gene product in wild type sample). Rp49 RNA was used as control to normalize the data.

**RNA interference.**

The day before transfection, 0.9 × 10^6^ S2 cells were transferred to a six-well culture dish 1 h prior to pretreatment with dsRNA. To knock down wild-type (endogenous) *mle* RNA while leaving mutant *mle*(ΔG) RNA intact, 30 μg/well of dsRNA (Ambion's MEGAscript protocol) covering the 94-amino-acid ΔG deletion was used. Green fluorescent protein (GFP) dsRNA (20 μg/well) was used as an RNA interference control. Cells were transfected 18 to 22 h later as described under “Dosage compensation” above.

**Plasmid construction.**

The N terminal Flag-tagged MLE recombinant clones were obtained using the strategies described below.

For the mutant missing both RNA-binding motifs (∆RB 1,2), the following PCR primers were designed to introduce both the deletion of the fragment from amino acid 1‑241 and the EcoRV site in the 5’ region of the *mle* gene:

RV/NtDSRMd oligo: CCATGGGATATCCTTGCCGCCGGA

KD-Rev oligo: GGCAGGAATACTAAGATAG

The PCR product obtained using the pFLC1mle plasmid (Drosophila Genome Research Center stock #RE21725) was cloned in the pTopo 2.1 vector (Invitrogen) and the EcoRV-XhoI fragment was substituted in the same sites of the Flag-mle/pBS subclone [35].

For the mutant missing the second RNA-binding motif (∆RB2), achieved by deleting amino acids 123-252, the two following oligos were annealed to provide StuI and HindIII overhangs (5’ and 3’ sites, respectively) to substitute in the same sites of the Flag-mle/pBS subclone [35]:

Forward: CCTATCGCCCTCTAAAAAAAAAGGATGAGCAGCTGAAGCCATATCCGGTGA

Reverse: AGCTTCACCGGATATGGCTTCAGCTGCTCATCCTTTTTTTTTAGAGGGCGATAGG

For the mutant missing the first RNA-binding motif (∆RB1), achieved by deleting amino acids 3-86, the following oligos with compatible SmaI (5’) and AvrII (3’) ends and spanning the region to be deleted (amino acids 3-86) were annealed and inserted in the Flag-pBS/mle clone [35]:

Forward: CCCATGGATTACGACGACGATGATAAATGGATGGCGGACCCT

Reverse: AGGGTCCGCCATCCATTTATCATCGTCGTCGTAATCCATGGG

To obtain the mutant missing the C-terminus glycine-rich motif (∆G), two oligos (see below) with PstI (5’) and NotI (3’) overhangs were annealed and subcloned in the Flag-mle/pBlueBac 4.5 clone (which contained the entire Flag-mle SmaI/BamHI fragment in the pBlueBac4.5 vector). The XbaI (provided by the pBlueBac 4.5 polylinker) and BamHI fragment was then subcloned in the pBS vector.

Forward: GCGTGAATCAGGAATTCTGCCGCACCAATCACGTCAGTTCAGTGACGGC

Reverse: GGCCGCCGTCACTGAACTGACGTGATTGGTGCGGCAGAATTCCTGATTCACGCTGCA

The SmaI-BamHI fragment from each recombinant pBS vectors was cloned in the pMK33/pMtHy vector [50]. Two oligonucleotides containing EcoRI and SacII sites at the 5′ end and BamHI and EcoRI sites at the 3′ end were annealed, and the dsDNA fragment was cloned into the EcoRI site of the pCasperhs83 vector. The SacII-BamHI fragment from each recombinant Flag tag-*mle* vector was subcloned in this modified pCasperhs83 vector.

All the final subcloned fragments were rechecked by sequencing.
